# An NLRP3 inflammasome inhibitor evoked dose-dependent anti-allodynia in the hindpaws of a rat model of chemotherapy-induced peripheral neuropathy

**DOI:** 10.1007/s10787-026-02360-w

**Published:** 2026-08-10

**Authors:** L. H. C. Kek, M. Z. Imam, O. Sriwatananukulkit, A. Kuo, K. Schroder, M. T. Smith

**Affiliations:** 1 Faculty of Health, Medicine and Behavioural Sciences, Centre for Integrated Preclinical Drug Development, School of Biomedical Sciences, The University of Queensland, St Lucia Campus, Brisbane, QLD 4072 Australia; 2 Institute for Molecular Bioscience, The University of Queensland, Brisbane, QLD Australia

**Keywords:** Chemotherapy induced peripheral neuropathy (CIPN), Cisplatin, Mechanical allodynia, Neuropathic pain, MCC950, NLRP3 inflammasome inhibitor, Pregabalin

## Abstract

Patients receiving chemotherapy for cancer treatment may develop chemotherapy-induced peripheral neuropathy (CIPN), a type of neuropathic (nerve) pain that is often difficult to treat. First-line analgesic/adjuvant agents recommended for the treatment of neuropathic pain often lack efficacy and/or evoke dose-limiting side-effects in patients with CIPN. Hence, there is a large unmet medical need for novel, well-tolerated analgesics for improving the relief of CIPN. As NLRP3 inflammasome activation is implicated in the pathobiology of CIPN, our aim was to assess the pain relief efficacy of a small molecule NLRP3 inflammasome inhibitor (MCC950), for the relief of CIPN in a rat model. Sprague–Dawley rats received four doses of cisplatin at once-weekly intervals. Temporal development of mechanical allodynia was documented in the bilateral hindpaws using von Frey filaments over a 4-week period after the first cisplatin dose. Rats with fully developed mechanical allodynia in the hindpaws received single oral doses of MCC950 (10–300 mg/kg), pregabalin (30 mg/kg) or vehicle. Hindpaw withdrawal thresholds were assessed pre-dose and at multiple times over a 4 h post-dosing period to produce PWT versus time curves. Single doses of MCC950 evoked dose-dependent anti-allodynia in the hindpaws, with the peak effect observed at 2–3 h post-dose. The mean effective dose 50% (ED_50_; 95% confidence intervals) of MCC950 was 56.2 mg/kg (21.8–150.7 mg/kg). On a molar basis, oral pregabalin was ~ 20% more potent than MCC950 in this model. In conclusion, NLRP3 inflammasome inhibitors are worthy of further investigation as novel treatments for CIPN.

## Introduction

For patients receiving potentially-curative chemotherapy treatment regimens including taxanes, vinca alkaloids, platinum derivatives and proteosome inhibitors, up to 90% may develop chemotherapy-induced peripheral neuropathy (CIPN), a type of neuropathic (nerve) pain that develops in a ‘stocking and glove’ distribution and that is a major dose-limiting side-effect (Bae et al. 2021; Colvin 2019; Han and Smith 2013; Maihofner et al. 2021). CIPN is often difficult to alleviate with analgesic/adjuvant agents recommended by the Neuropathic Pain Special Interest Group of the International Association for the Study of Pain (Soliman et al. 2025). When CIPN pain is severe, a switch to less effective chemotherapy drugs may be needed or patients may choose to discontinue treatment, potentially increasing cancer-associated morbidity and mortality (Gewandter et al. 2020; Seretny et al. 2014). Hence, the large unmet medical need for novel, efficacious and well-tolerated analgesic/adjuvant agents for relief of CIPN is driving research on its pathophysiology aimed at identifying novel targets to use in analgesic drug discovery programs.

One such target is the NLRP3 inflammasome, a macromolecular protein complex comprising a sensor (NLRP3), an adaptor (apoptosis-associated speck-like protein, ASC) and an effector (caspase-1) (reviewed in (Coll and Schroder 2025)). NLRP3 is a tripartite protein containing an amino-terminal pyrin domain (PYD), a central NACHT domain and a carboxy-terminal leucine-rich repeat (LRR) domain (reviewed in (Chen and Smith 2023)). ASC comprises two domains, PYD and the caspase recruitment domain (CARD), and is essential for bridging interactions between NLRP3 upstream, and the caspase-1 protease downstream, to form an ‘ASC speck’ structure during signalling (Coll and Schroder 2025). ASC forms fibrils to grow the superstructure of the NLRP3 inflammasome complex to amplify signalling (Coll and Schroder 2025). Interestingly, the orally active small molecule NLRP3 inflammasome inhibitor, MCC950, binds to a pocket in the NACHT domain, and locks NLRP3 in an inactive conformation (Coll and Schroder 2025). Importantly, MCC950 does not inhibit inflammasomes other than the NLRP3 inflammasome (Coll and Schroder 2025).

Multiple sterile and microbial stimuli activate the NLRP3 inflammasome via a two-step process comprising priming (Signal 1) (He et al. 2016) and activation (Signal 2) (Zhang et al. 2020). Priming is induced by nuclear factor-kappa B-activating pathways such as those induced by recognition of pathogen-associated molecular patterns (PAMPs) by pattern recognition receptors such as toll-like receptors (Chen and Smith 2023). Signal 2 comprises many PAMPs and damage-associated molecular patterns (DAMPs) including crystals, particles, extracellular adenosine triphosphate, chemotherapy agents and debris from cancer cells (Chen and Smith 2023). The net effect is upregulated expression of NLRP3 and caspase-1 activation to convert inactive pro-interleukin-1β (pro-IL-1β) and pro-IL-18 to the corresponding active, pro-inflammatory/pronociceptive cytokines, IL-1β and IL-18, that may sensitise nociceptors (Chen and Smith 2023).

In a paclitaxel-induced rat model of CIPN, there was elevated expression of NLRP3, caspase-1, and IL-1β in the sciatic nerve and lumbar dorsal root ganglia (DRGs) which was accompanied by increased generation of reactive oxygen species and mitochondrial damage in the sciatic nerve, which contributed further to activation of NLRP3 (Jia et al. 2017). In an oxaliplatin-induced rat model of CIPN, there was increased expression of NLRP3 and caspase-1 in the spinal dorsal horn and once-daily intrathecal dosing for 25 days with the NLRP3 inhibitor, MCC950, inhibited NLRP3 activation (Wahlman et al. 2018). Interestingly, the NLRP3 inflammasome is also implicated in the pathobiology of CIPN induced by cisplatin, a chemotherapy agent used to treat a range of solid cancers (reviewed in (Domingo et al. 2022)). Cisplatin is associated with persistent markers of inflammation from the innate immune system (Domingo et al. 2022). Of the NOD-like receptors, the NLRP3 inflammasome appears to contribute most to cisplatin induced CIPN (Domingo et al. 2022). As the NLRP3 inhibitor, MCC950, has an oral bioavailability of 68% in mice (Coll et al. 2019, 2015; Tapia-Abellan et al. 2019) and it crosses the blood–brain-barrier (Chen et al. 2022a), we hypothesised that oral MCC950 may be efficacious for the relief of fully developed pain behaviour in a cisplatin-induced rat model of CIPN.

## Methods

### Materials

Cisplatin injection vials (100 mg/ 100 mL) were from The Wesley Hospital Pharmacy (Brisbane, Australia). The NLRP3 inflammasome inhibitor, MCC950 (N-(1,2,3,5,6,7-Hexahydro- *s*-indacen-4-ylcarbamoyl)-4-(2-hydroxy-2-propanyl)-2-furansulfonamide) as the sodium salt (purity 99.75%), was from MedChemExpress (Monmouth Junction, New Jersey, USA) and supplied by their Australian agent, Focus Bioscience (Sydney, Australia). Pregabalin was from Toronto Research Chemicals INC (North York, Ontario, Canada). Sterile water for injection (WFI) ampoules and sterile normal saline ampoules, were from Pfizer Inc. (Sydney, NSW, Australia). Topical antibiotic powder was from Apex Laboratories Pty Ltd (Somersby, NSW, Australia).

### Animals

Ethics approval was from The University of Queensland Animal Ethics Committee (Approval number: 2023/AE000699) and this work was undertaken in compliance with the National Health and Medical Research Council’s Australian Code for the Care and Use of Animals for Scientific Purposes (8th edition, 2013). Male Sprague–Dawley rats (n = 17; 160 to 180 g) were from Ozgene-ARC (Perth, Australia). Rats were housed in groups of two to three in individually ventilated cages in a purpose-built, temperature-controlled holding facility (21 ± 2 °C, mean ± SD) with a fixed 12 h/12 h light–dark cycle at the Centre for Integrated Preclinical Drug Development. Rats had free access to rodent chow and water, and they were acclimatized for at least three days before initiation of experimentation. Rodent hutches, chewsticks, and Kimwipes were used for environmental enrichment.

### Induction of cisplatin-induced CIPN in rats

CIPN was induced by intraperitoneal (IP) injection of cisplatin (3 mg/kg) once weekly for four weeks on days 0, 7, 14 and 21 with a cumulative dose of 12 mg/kg. This cisplatin dosing regimen evoked temporal development of mechanical hypersensitivity in the bilateral hindpaws which was fully developed by day 21 after the first dose of cisplatin and it persisted for at least 42 days. At 5 min before each cisplatin injection, rats received a subcutaneous injection of sterile saline (2 mL) to hyperhydrate the animals and avoid nephrotoxicity.

### General health monitoring

For each rat, body weight was measured and urinalysis was performed at once-weekly intervals commencing prior to the first cisplatin dose. Haematocrit was assessed once fortnightly on the day before cisplatin injection. Baseline von Frey paw withdrawal thresholds (PWTs) were measured on the day prior to each cisplatin injection (Days -1, 6, 13, 20). For haematocrit measurements, blood samples were taken from the tail vein and placed into labelled haematocrit tubes (75 mm; Drummond Scientific Co., Broomall, Pennsylvania, USA). Using a haematocrit rotor set at 10,000 g (Damon/IEC division, IEC MB centrifuge, Micro Haematocrit; Hawksley & Sons Ltd, West Sussex, UK), blood samples were centrifuged for 5 min at room temperature. In male Sprague–Dawley rats, the normal range of values for haematocrit is 34–57% (Han et al. 2014). For urinalysis, urine dipsticks (Multi reagent strips 10SG, Livingstone) were used to monitor renal function, as cisplatin frequently causes nephrotoxicity.

### Assessment of von Frey Paw withdrawal thresholds in the hindpaws

On the day prior to each cisplatin injection, rats were housed individually in cages with wire mesh floors and given at least 30 min to acclimatise to the testing environment. Calibrated Von Frey filaments that delivered graded forces of 2, 4, 6, 8, 10, 12, 16, 18 and 20 g were applied to each of the hindpaws and PWTs were assessed using the up-down method. Specifically, the 6 g filament was applied first to the plantar surface of the hindpaw for 3 s until the filament buckled slightly. If no response was evoked after three seconds of applying the filament, the next filament in an ascending sequence (2, 4, 6, 8, 10, 12, 14, 16, 18, and 20 g) was used until hindpaw withdrawal was elicited. If no response was elicited by the 20 g filament, rats were assigned a measurement of 20 g arbitrarily. Conversely, if application of the 6 g filament evoked a hindpaw withdrawal response, the next filament in the descending sequence was used until the filament that delivered the lowest force that evoked a hindpaw withdrawal response within three seconds of filament application, was identified. Hindpaw PWTs were measured in the left hindpaw first, then in the right hindpaw five min later. There were three repetitions of this sequence, with a 5-min interval between each sequence. The mean of the three measurements for each hindpaw served as the baseline PWT on each testing occasion. For post-dosing, one measurement per time point was performed.

### Treatment groups

Commencing on day 28 after the first dose of cisplatin, rats exhibiting fully developed mechanical hypersensitivity in the bilateral hindpaws were randomized to receive a single oral dose of MCC950 in approximate half-log units (10, 30, 50, 100 or 300 mg/kg) to define the dose–response relationship, oral pregabalin (30 mg/kg) as the positive control, or oral vehicle (negative control). Dosing solutions were prepared using sterile WFI as the vehicle. Each rat received up to five single doses of the test or reference compound or vehicle according to a ‘washout’ protocol, such that there were at least two days of washout between successive doses. Just prior to dosing on each testing occasion, PWTs in the bilateral hindpaws were measured using von Frey filaments and then at 0.25, 0.5, 0.75, 1, 1.25, 1.5, 2, 3, and 4 h post-dose. Von Frey PWTs in the bilateral hindpaws of CIPN-rats were assessed by a tester blinded to treatment group to avoid unconscious bias.

### Adverse effects

A standardized set of approximately 40 clinical signs in categories including (i) skin and fur (piloerection, lack of grooming, fur loss, irritation or sores), (ii) eyes and mucus membranes (cloudy eyes, ocular or nasal discharge, diarrhoea etc.), (iii) respiratory and circulatory function (respiratory rate, gasping etc.), (iv) gait and posture (hunched posture, walking on tiptoe etc.), (v) behaviour (reduced/increased activity, circling, repetitive sniffing, Straub tail etc.), (vi) clonic tremors (tremors, myoclonic jerks, wet dog shakes etc.), (vii) tonic tremors (head and body rigidly arched etc.). The observations graded as mild, moderate and severe, if any, were used to assess adverse effects.

### Statistical analysis

For the baseline PWT versus time curve data showing the temporal development of mechanical hypersensitivity in the bilateral hindpaws of CIPN-rats, a one-way ANOVA with Dunnett’s multiple comparisons test was performed using GraphPad Prism (v11.0.0). Mean (± SEM) PWT vs time curves for each oral dose of MCC950, pregabalin, and vehicle were also generated. For each individual animal, the increase in PWT above baseline values was calculated by subtracting the pre-dosing PWT value from each post-dosing PWT value to give ΔPWT versus time curves. Trapezoidal integration as implemented in GraphPad Prism (v11.0.0) was utilised to estimate the area under the ΔPWT versus time curve values (ΔPWT AUCs) for individual animals. Dose–response curves were generated by plotting mean (± SEM) ΔPWT AUC values versus log dose. Nonlinear regression in GraphPad Prism (v11.0.0) was used to determine the ED_50_ (95% confidence intervals) for oral MCC950 in CIPN-rats. A two-way ANOVA with Dunnett’s multiple comparisons test was used to compare mean (± SEM) ΔPWT AUC values for each dose level relative to that for the vehicle-treated group. For one-way ANOVA, F values are expressed as *F*_df_ of treatment, residual. For 2-way ANOVA, F values are expressed as *F*_df_ of treatment, time, interaction/residual. An unpaired t-test was used to compare the ΔPWT AUC values for single oral doses of MCC950 at 100 mg/kg and pregabalin at 30 mg/kg. The statistical significance criterion was *p* < 0.05.

## Results

### General health

**Fig. 1:**
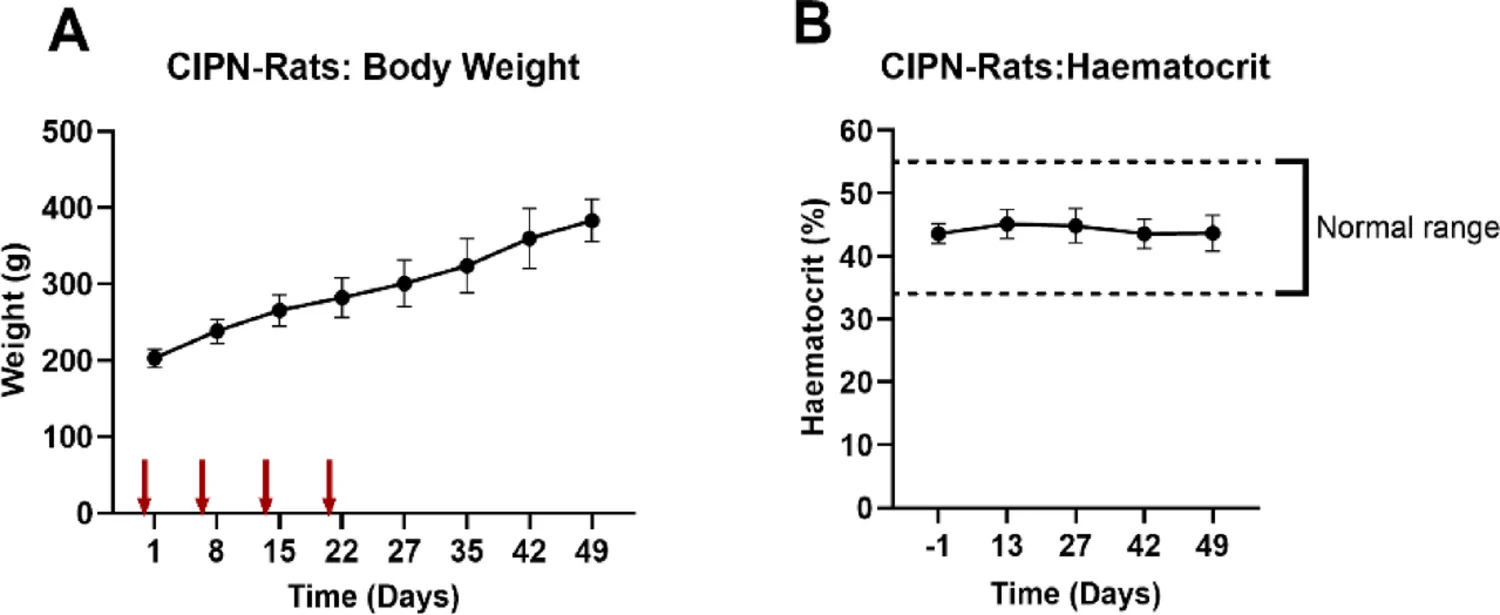
Mean (± SD) body weight (**A**) and haematocrit (**B**) values for cisplatin-induced CIPN rats (n = 17) for the 49-day study period. Body weight was measured once weekly. Haematocrit (normal range 34%-57%) was measured once fortnightly. Baseline measurements were prior to the first dose of cisplatin. Cisplatin injections are indicated by the brown arrows

The mean (± SD) body weight of rats increased with time during the 49-day study period (Fig. 1A), indicative of good general health and consistent with expectations and previous work from our laboratory where we reported body weight data for cisplatin-treated versus vehicle-treated rats during optimisation of this model (Han et al. 2014). The mean (± SD) haematocrit levels remained within the normal range (34–57%) (Fig. 1B) as did the indices of kidney function measured in the collected urine samples (Table 1) that spanned the period during which the anti-allodynic effects of MCC950 were evaluated in these CIPN-rats. Together, these data are aligned with our previous findings for this model (Han et al. 2014) and they show that the general health of animals was satisfactory throughout the 49-day experimental period (Fig. 1).

**Table 1:** Urinalysis data for rats prior to initiation of the cisplatin dosing regimen (day -1) and during the cisplatin dosing regimen (days 0–27)

Group (n = 17)	Glucose	Bilirubin	Ketone	Specific gravity	Erythrocytes	pH	Protein	Urobilinogen	Nitrite	Leukocytes
Normal rats (day -1)	–	–	–	1.01	–	7.91	–	–	–	–
CIPN rats (days 0–27)	–	–	–	1.01	–	7.34	–	–	–	–

Not detected (–)

Importantly, the urinalysis data for rats during the cisplatin dosing period (Day -1 to Day 27) remained within the normal range (Table 1) mirroring previous data from our laboratory for this model (Han et al. 2014). The parameters measured were glucose, bilirubin, ketone, erythrocytes, protein, urobilinogen, nitrite and leukocytes (Table 1). The mean specific gravity (1.01) of the rat urine samples also remained within the normal range (McClure 1999). Although there was a small difference in mean urine pH values between that measured prior to the first dose of cisplatin at 7.91 and that for rats that had received up to 4 doses of cisplatin at 7.34, all urine pH values were within the normal range (7.0–8.0) for rodents (McClure 1999).

### Temporal development of mechanical allodynia in the hindpaws of CIPN rats

**Fig. 2:**
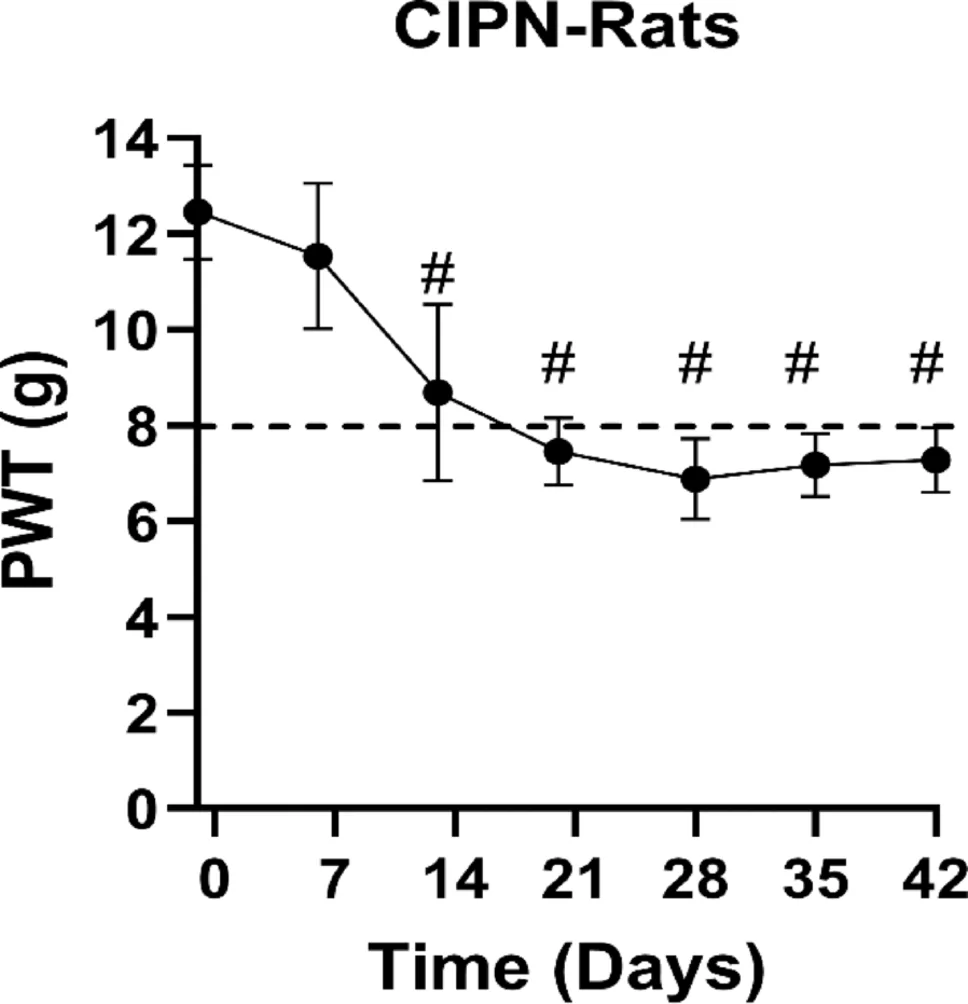
Temporal decrease in mean (± SD) paw withdrawal thresholds (PWTs) in the bilateral hindpaws of rats (n = 17) after initiation of the cisplatin dosing regimen showing that mechanical hypersensitivity was fully developed by day 21 (PWTs ≤ 8 g). Mean (± SD) PWTs remained ≤ 8 g from Day 21 until Day 42. Statistical analysis was performed using one-way ANOVA followed by Dunnett’s multiple comparisons test (F_6, 110_ = 69.28, *p* < 0.0001). #indicates statistical significance (*p* < 0.0001) relative to the pre-cisplatin baseline PWT values

There was temporal development of mechanical hypersensitivity in the bilateral hindpaws of CIPN-rats such that the mean (± SD) PWTs decreased from 12.5 g (± 1.0) on Day − 1 to 7.5 g (± 0.7) on Day 21, indicating that mechanical hypersensitivity was fully developed (PWTs ≤ 8 g) (Fig. 2). This observation is consistent with earlier research from our laboratory showing that mechanical allodynia in this CIPN rat model is fully developed by day 28 after the first dose of cisplatin (Han et al. 2014). Importantly, the extent of mechanical allodynia in the bilateral hindpaws was stable for the 42-day study period as confirmed by mean (± SD) PWTs remaining ≤ 8 g from day 21 to day 42, consistent with previous work from our laboratory (Han et al. 2014).

### Pain relief efficacy of MCC950 relative to pregabalin and vehicle in CIPN rats

**Fig. 3:**
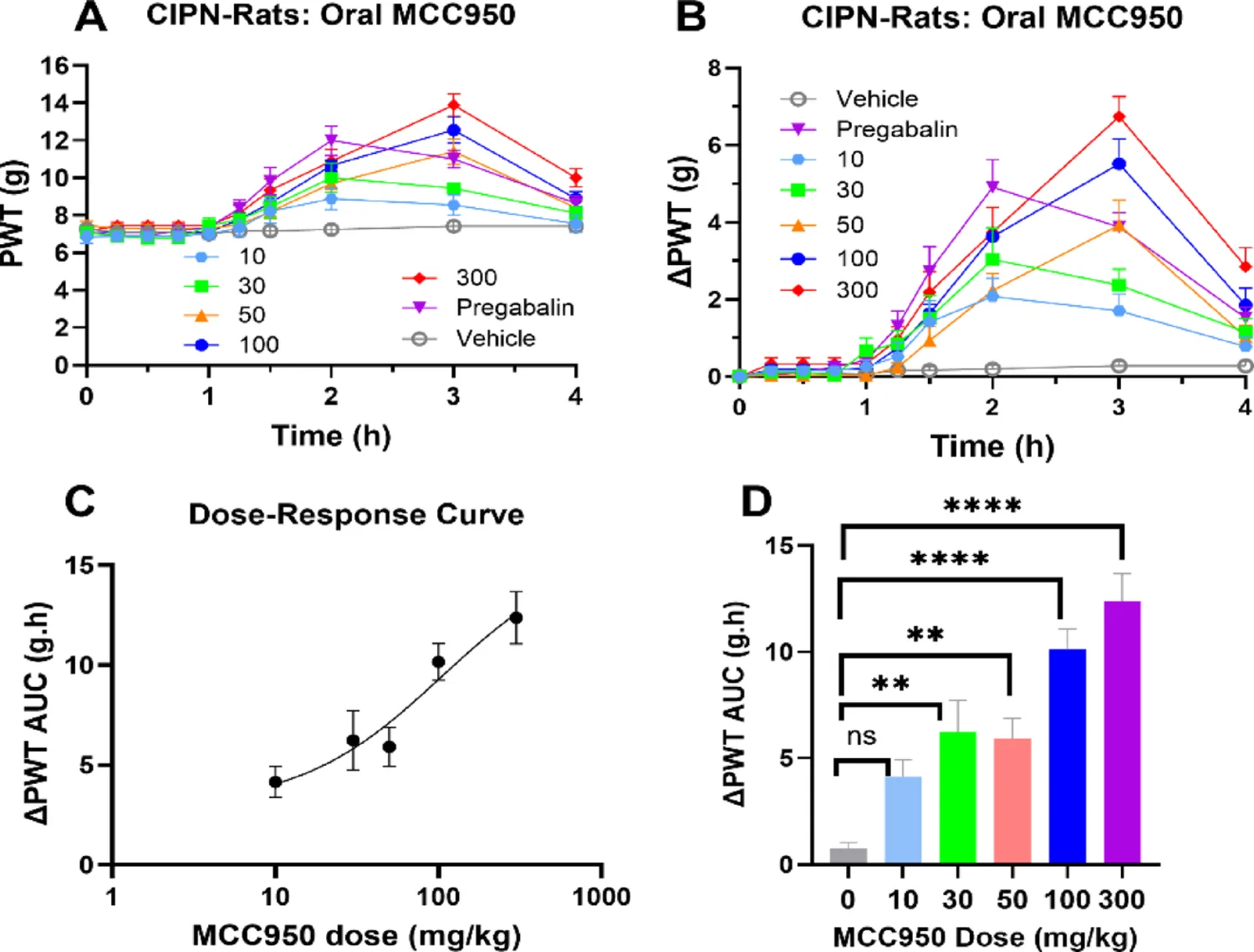
**A** Mean (± SEM) paw withdrawal threshold (PWT) (g) versus time curves following administration of single oral doses of MCC950 (10, 30, 50, 100, and 300 mg/kg) relative to oral pregabalin (30 mg/kg) and oral vehicle in CIPN-rats. **B** Mean (± SEM) increase in PWT above pre-dosing PWT values (ΔPWTs) versus time curves evoked by single oral doses of MCC950 (10, 30, 50, 100, and 300 mg/kg), oral pregabalin (30 mg/kg) or oral vehicle in CIPN-rats. **C** Dose–response curve for oral MCC950 in CIPN rats. Data points are the mean (± SEM) ΔPWT AUC values for each dose. The mean ED_50_ (95% confidence intervals) estimated using nonlinear regression was 56.2 mg/kg (21.8 to 150.7 mg/kg). **D** Single oral doses of MCC950 at 30, 50, 100 and 300 mg/kg (but not 10 mg/kg) evoked significantly greater (*p* < 0.05) anti-allodynic responses (ΔPWT AUC values) relative to the corresponding response evoked by vehicle. **** and ** indicate statistical significance (*p* < 0.0001 and *p* < 0.01 respectively) relative to the vehicle whereas ns indicates no significant difference (*p* ≥ 0.05)

Single oral doses of MCC950 (10, 30, 50, 100 or 300 mg/kg) evoked dose-dependent anti-allodynia in the bilateral hindpaws of CIPN-rats (Fig. 3A). The corresponding mean (± SEM) ΔPWT versus time curves are shown in Fig. 3B. Inspection of the mean (± SEM) PWT and ΔPWT versus time curves (Fig. 3A, B respectively), shows that the mean onset of action for both MCC950 and pregabalin was ~ 1.5 h. For the approximately equi-effective doses of oral MCC950 (100 mg/kg) and oral pregabalin at 30 mg/kg, the mean time of peak effect was 2 h for pregabalin compared with 3 h for MCC950 (Fig. 3A, B). For these doses of MCC950 and pregabalin, the mean (± SEM) duration of action was 3–4 h.

The mean (± SEM) ΔPWT AUC values were plotted against log dose to generate a dose–response curve for MCC950 (Fig. 3C). The mean ED_50_ (95% confidence intervals) estimated using nonlinear regression was 56.2 mg/kg (21.8 to 150.7 mg/kg). Importantly, single oral doses of vehicle did not evoke significant anti-allodynia in the bilateral hindpaws, consistent with expectations and with our previous findings using this rat model of CIPN (Han et al. 2014). Additionally, for the dose range investigated, single oral doses of MCC950 did not evoke overt behavioural side-effects in CIPN-rats, similar to oral doses of vehicle.

**Fig. 4:**
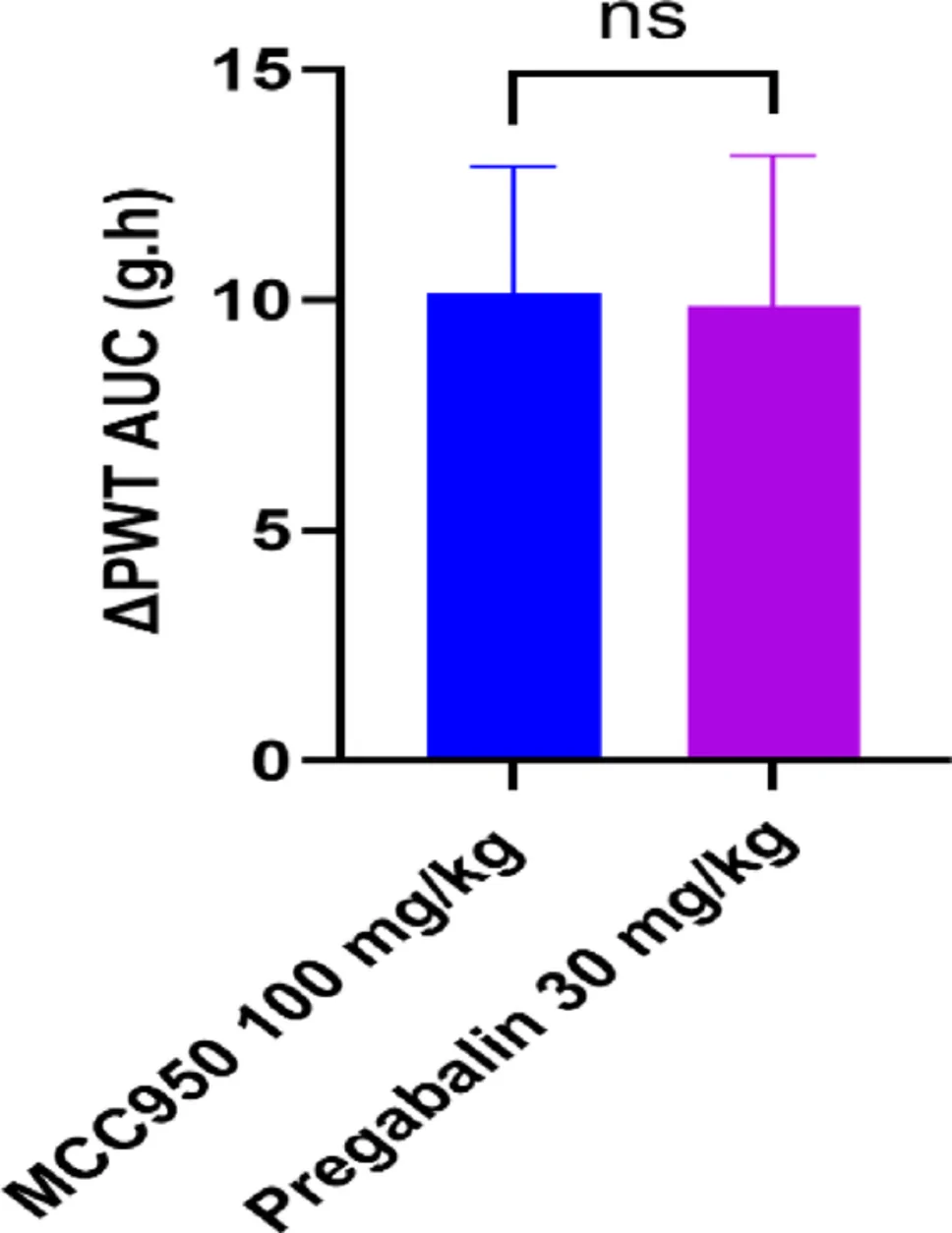
Mean (± SEM) area under the ΔPWT versus time curves (ΔPWT AUC values) for oral MCC950 (100 mg/kg; 234.5 µmol/kg; blue, n = 9) and oral pregabalin (30 mg/kg; 188.4 µmol/kg; purple, n = 10) in CIPN-rats. The mean (± SEM) ΔPWT AUC values did not differ significantly (*p* > 0.05) between the treatments showing that a single oral dose of pregabalin is approximately 1.2-fold more potent than a single oral dose of MCC950

Inspection of Fig. 4 shows that the mean (± SEM) ΔPWT AUC for oral MCC950 at 100 mg/kg is not significantly different (P > 0.05) from that for oral pregabalin at 30 mg/kg. As the molecular weight of MCC950 is approximately 2.7-fold higher than that of pregabalin, these data show that oral pregabalin is approximately 1.2-fold more potent than oral MCC950 on a molar basis, in this model.

## Discussion

**Fig. 5:**
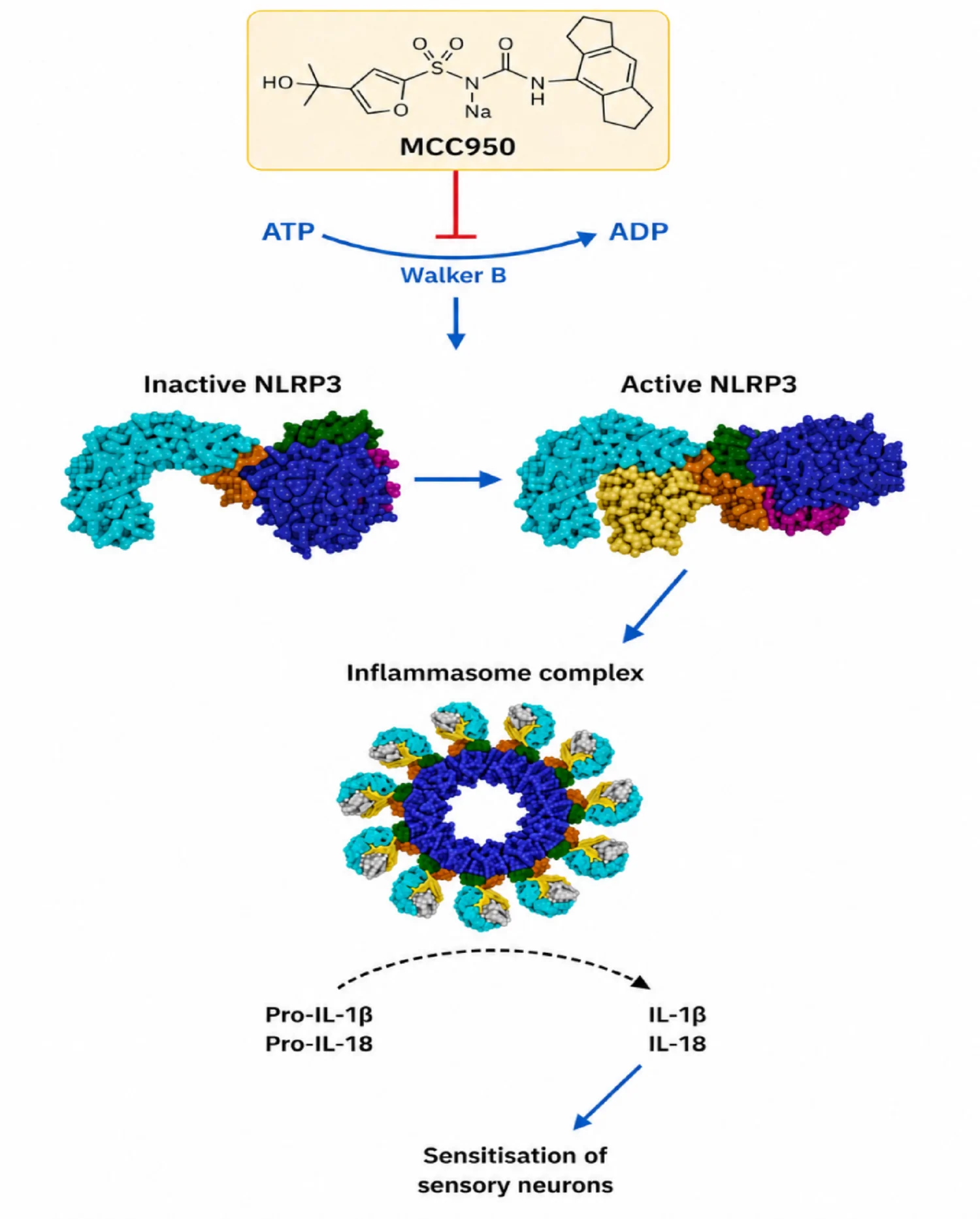
Schematic diagram showing that MCC950 inhibits the ATPase that catalyses the conversion of NLRP3 from its inactive to its active state, thereby inhibiting inflammasome formation. This results in reduced secretion of the proinflammatory/pronociceptive cytokines, IL-1β and IL-18, into the cellular milieu that would otherwise sensitise sensory neurons. The net result is decreased ectopic firing of sensitised sensory neurons which in turn would reduce central sensitisation in the CNS, a key pathobiological mechanism that contributes to the development and maintenance of neuropathic pain states such as CIPN

In the present study in rats with cisplatin-induced CIPN exhibiting fully developed mechanical allodynia in the bilateral hindpaws, single oral doses of the small molecule NLRP3 inflammasome inhibitor, MCC950 (10–300 mg/kg), evoked dose-dependent anti-allodynia and there were no discernible adverse effects at the doses tested. Our findings are aligned with a previous report by others implicating a role for NLRP3 inflammasome activation in the pathobiology of cisplatin-induced CIPN (reviewed in (Domingo et al. 2022)). As MCC950 crosses the blood–brain-barrier (Chen et al. 2022a), it would be expected to inhibit NLRP3 inflammasome activation not only in macrophages in the peripheral nervous system (PNS) but also in microglia in the central nervous system (CNS). As MCC950 prevents the conversion of inactive NLRP3 to its active state (Fig. 5), this in turn would reduce release of the pro-inflammatory/pronociceptive cytokines, IL-1β and IL-18 into the cellular milieu resulting in decreased sensitisation of sensory neurons in both the PNS and the CNS to decrease ectopic firing of sensitised sensory neurons and evoke pain relief (Chen and Smith 2023). In support of this notion, Martinez-Martel et al. (2025) showed that the progressive reversal of hindpaw hypersensitivity in CIPN-mice by MCC950 given twice-daily at 10 mg/kg for 3 consecutive days, was underpinned by inhibition of NLRP3 inflammasome activation in the sciatic nerve (PNS), amygdala and hippocampus (CNS). Furthermore, MCC950 normalized the otherwise overexpression of the putative pain biomarker, phospho-ERK 1/2, in the sciatic nerve and apoptotic responses in the sciatic nerve and amygdala (Martinez-Martel et al. 2025).

Our findings also extend work by others that showed a single IP dose of MCC950 at 10 mg/kg evoked partial relief of mechanical and thermal hypersensitivity in the bilateral hindpaws of a mouse model of CIPN induced with paclitaxel (Martinez-Martel et al. 2025). Interestingly in the same study, twice-daily IP doses of MCC950 at 10 mg/kg for 3 consecutive days, progressively alleviated both mechanical and thermal hypersensitivity in the bilateral hindpaws of these mice (Martinez-Martel et al. 2025). In other work using the chemotherapy agent, vincristine, to induce CIPN in mice (Starobova et al. 2025), once-daily IP administration of MCC950 at 10–20 mg/kg for 4-weeks according to a prevention protocol, evoked dose-dependent prevention of the development of mechanical hypersensitivity in the bilateral hindpaws. Collectively, findings in rodent models of CIPN induced with three different chemotherapy agents (cisplatin, vincristine and paclitaxel), further implicate a role for NLRP3 inflammasome activation in the pathobiology of CIPN.

Additional insight into the mechanisms through which cisplatin induces peripheral neuropathy comes from work in cultured dissociated adult mouse DRG neurons, which are rich in mitochondria. DRG neurons were incubated for 24 h with a sub-lethal concentration of cisplatin (10 µM), resulting in elevated concentrations of reactive oxygen species (ROS), depletion of mitochondrial DNA and impaired mitochondrial respiration (Gorgun et al. 2017). These DRG neuronal insults would be expected to activate the NLRP3 inflammasome (Domingo et al. 2022), triggering the production of the proinflammatory/pronociceptive cytokines, IL-1β and IL-18, resulting in sensitization of DRG neurons, as has been reported in mouse models of inflammatory pain (Silva Santos Ribeiro et al. 2023). Consistent with this notion, vincristine-induced CIPN was driven by NLRP3 inflammasome activation and increased release of IL-1β from macrophages in sciatic nerves and DRGs but not the spinal cord of mice (Starobova et al. 2021). Additionally, vincristine-induced mechanical hypersensitivity in the hindpaws of these mice was significantly reduced by treatment with MCC950 at 20 mg/kg concurrently with vincristine, or in vincristine-treated mice lacking NLRP3 signaling pathway components (Starobova et al. 2021). In other work in a paediatric patient-derived xenograft mouse model of acute lymphoblastic leukemia (ALL), treatment of mice with MCC950 at 15 mg/kg/day IP for 4-weeks concurrently with once-weekly administration of lower doses of vincristine (0.1–0.5 mg/kg IP), attenuated the development of mechanical hypersensitivity in the hindpaws (Starobova et al. 2025). However, this same MCC950 dosing regimen lacked efficacy for the relief of mechanical hypersensitivity in the hindpaws of mice administered higher vincristine dosing regimens (1 and 1.25 mg/kg IP once weekly for 4 weeks) (Starobova et al. 2025). Based on our findings herein that single oral doses of MCC950 at 100 mg/kg were required to completely relieve hindpaw hypersensitivity in a rat model of fully established cisplatin-induced CIPN, it is plausible that MCC950 at doses larger than 15 mg/kg/day IP may be required to alleviate hindpaw hypersensitivity in the high dose vincristine induced patient-derived mouse model of ALL; this remains for future investigation.

In other rodent neuropathic pain models such as a mouse model of painful diabetic neuropathy (PDN), single doses of MCC950 (3 and 10 mg/kg IP) evoked dose-dependent partial attenuation of mechanical hypersensitivity in the bilateral hindpaws (Chen et al. 2022b). Investigation of the efficacy of larger doses of MCC950 is needed in future work. In the same study, lumbar DRG and lumbar spinal cord expression levels of IL-1β in MCC950-treated PDN-mice were lower than the corresponding expression levels in vehicle-treated PDN-mice, consistent with NLRP3 inhibition by MCC950, resulting in attenuation of the release of the proinflammatory cytokines, IL-1β and IL-18 (Chen et al. 2022b). The congruence between decreased IL-1β expression levels in the lumbar DRGs and lumbar spinal cords of MCC950-treated PDN-mice, and attenuation of mechanical allodynia in the bilateral hindpaws of these mice, further implicates a key role for NLRP3 inflammasome signalling in the pathobiology of PDN (Chen et al. 2022b), another type of neuropathic pain that is notoriously difficult to treat. Additionally, in the chronic constriction injury (CCI) induced rat model of neuropathic pain exhibiting fully developed hindpaw hypersensitivity, once-daily intrathecal administration of MCC950 at 30 µg/kg/day for 14 consecutive days partially alleviated mechanical allodynia in the hindpaws, implicating a pathological role for NLRP3 inflammasome signalling in this condition (Kui et al. 2025). In other work in a mouse model of trigeminal neuralgia (another type of neuropathic pain), single doses of MCC950 (5 mg/kg IP) evoked partial relief of mechanical allodynia in the affected area of the face (Ren et al. 2021). In the Experimental Autoimmune Encephalomyelitis (EAE) mouse model of multiple sclerosis-associated neuropathic pain, female mice with fully developed mechanical allodynia in the bilateral hindpaws were administered oral MCC950 at 50 mg/kg/day for 21 consecutive days (Khan et al. 2018). This dosing regimen progressively reversed mechanical allodynia in the hindpaws, implicating the NLRP3 inflammasome in the pathobiology of central neuropathic pain (Khan et al. 2018). This notion was further supported by work in a rat model of central post-thalamic hemorrhagic stroke pain, where pretreatment of animals with NLRP3 siRNA (but not missense) or a single intra-thalamic injection of MCC950 (but not vehicle) prevented the development of mechanical hypersensitivity in the hindpaws of these animals (Shi et al. 2023).

As pregabalin is a first-line analgesic/adjuvant agent recommended for the relief of neuropathic pain by the Neuropathic Pain Special Interest Group of the International Association for the Study of Pain (Soliman et al. 2025), it was used herein as the positive control analgesic agent. Interestingly, we found that single oral doses of pregabalin at 30 mg/kg (188.4 µmol/kg) were equi-effective with single oral doses of MCC950 at 100 mg/kg (234.5 µmol/kg) for the relief of mechanical allodynia in the bilateral hindpaws of CIPN-rats. This means that on a molar basis, oral pregabalin was approximately 20% more potent than oral MCC950 for evoking anti-allodynia in cisplatin-induced CIPN-rats. The pain relief efficacy of pregabalin for evoking anti-allodynia in the hindpaws of CIPN-rats herein is aligned with similar findings in multiple other rodent models of neuropathic pain (Kuo et al. 2021; Nozawa et al. 2022). The mechanism through which pregabalin attenuates mechanical allodynia in the hindpaws of CIPN-rats differs from that of MCC950, as pregabalin binds to the α2δ subunit of voltage-gated calcium channels in presynaptic nerve terminals to decrease the release of excitatory neurotransmitters at multiple levels of the central nervous system including the dorsal horn of the spinal cord (Mangaiarkkarasi et al. 2015). This suggests that combined dosing of an NLRP3 inflammasome inhibitor with pregabalin may produce pain relief synergy, but this remains to be assessed.

In the clinical setting, analgesic/adjuvant agents are administered chronically for the relief of CIPN. Hence, future work beyond the scope of that described herein will be directed at assessing the extent to which the pain relief evoked by oral MCC950 administered chronically, is maintained for a prolonged period. In previous work from our laboratory (Khan et al. 2018), oral MCC950 alleviated mechanical allodynia in the hindpaws of female EAE-mice and so it is unlikely that there are sex-related differences in the anti-allodynic effects of MCC950 in this rat model of CIPN.

### Conclusion

In conclusion, single oral doses of the NLRP3 inflammasome inhibitor, MCC950, evoked dose-dependent anti-allodynia in the cisplatin-induced rat model of CIPN with an ED_50_ of 56.2 mg/kg. At the doses tested, MCC950 was devoid of discernible adverse behavioural effects. The extent and duration of anti-allodynia evoked by a single oral dose of MCC950 at 100 mg/kg was not significantly different from that evoked by a single oral dose of pregabalin at 30 mg/kg in CIPN rats showing that on a molar basis pregabalin is ~ 1.2-fold more potent than MCC950. Our findings showing that single oral doses of MCC950 completely alleviated mechanical allodynia in the hindpaws of CIPN-rats when administered according to an intervention protocol, suggest that NLRP3 inflammasome inhibitors are worthy of further investigation as potential novel pain therapeutics for the relief of established CIPN.

## Data Availability

The data are available upon request.
